# OPTIMAL HEALTH PROFESSIONAL STAFFING OF PRIMARY CARE AND GERIATRIC PRACTICES SERVING FRAIL OLDER ADULTS

**DOI:** 10.1093/geroni/igz038.254

**Published:** 2019-11-08

**Authors:** Karen Donelan, Joanne Spetz

**Affiliations:** 1 Massachusetts General Hospital, Boston, Massachusetts, United States; 2 University of California, San Francisco, San Francisco, California, United States

## Abstract

This symposium will include 3 papers that provide critical interprofessional and interdisciplinary perspectives on our work to understand and measure staffing in health care teams caring for older adults, and frail older adults. The Health Teams for Frail Elders project was funded by the Gordon and Betty Moore Foundation from August 2016 to October 2018. Dr. Karen Donelan, project Principal Investigator, will chair the session, providing a brief project overview of project aims and activities. A survey and health services researcher, Dr. Donelan will set the context for this large scale project. Dr. Barbara Roberge, a geriatric nurse practitioner who established one of the first senior health programs in the nation along with Dr. Kenneth Minaker at Mass General Hospital, was our primary care and nursing lead on our site visits. She will talk about the care settings we visited, her development of a site assessment tool that covered a range of frail elder needs, and will summarize professional roles and staffing observed within different site types. Dr. Julie Berrett-Abebe, a junior investigator on our team (PhD 2017), will present a paper on the competencies and roles of social workers and community health workers in primary and geriatric practices, as well as the roles of community health workers. Dr. David Auerbach, a national expert in health policy and workforce analysis, will present 4 models of staffing of practices, demonstrating efficiencies in optimizing services for frail elders while minimizing costs.Dr. Joanne Spetz will be the discussant of cross-cutting themes.

